# Interim data monitoring to enroll higher-risk participants in HIV prevention trials

**DOI:** 10.1186/1471-2288-9-44

**Published:** 2009-06-23

**Authors:** Vera Halpern, Orikomaba Obunge, Folasade Ogunsola, Sakiru Otusanya, John Umo-Otong, Chin-Hua Wang, Neha Mehta

**Affiliations:** 1 Family Health International, Research Triangle Park, NC, USA; 2 Department of Medical Microbiology and Parasitology, University of Port Harcourt Teaching Hospital, Port Harcourt, Nigeria; 3 Department of Medical Microbiology and Parasitology, College of Medicine, University of Lagos, Lagos, Nigeria

## Abstract

**Background:**

Lower-than-expected incidence of HIV undermines sample size calculations and compromises the power of a HIV prevention trial. We evaluated the effectiveness of interim monitoring of HIV infection rates and on-going modification of recruitment strategies to enroll women at higher risk of HIV in the Cellulose Sulfate Phase III study in Nigeria.

**Methods:**

We analyzed prevalence and incidence of HIV and other sexually transmitted infections, demographic and sexual behavior characteristics aggregated over the treatment groups on a quarterly basis. The site investigators were advised on their recruitment strategies based on the findings of the interim analyses.

**Results:**

A total of 3619 women were screened and 1644 enrolled at the Ikeja and Apapa clinics in Lagos, and at the Central and Peripheral clinics in Port Harcourt. Twelve months after study initiation, the overall incidence of HIV was less than one-third of the pre-study assumption, with rates of HIV that varied substantially between clinics. Due to the low prevalence and incidence rates of HIV, it was decided to close the Ikeja clinic in Lagos and to find new catchment areas in Port Harcourt. This strategy was associated with an almost two-fold increase in observed HIV incidence during the second year of the study.

**Conclusion:**

Given the difficulties in estimating HIV incidence, a close monitoring of HIV prevalence and incidence rates during a trial is warranted. The on-going modification of recruitment strategies based on the regular analysis of HIV rates appeared to be an efficient method for targeting populations at greatest risk of HIV infection and increasing study power in the Nigeria trial.

**Trial Registration:**

The trial was registered with the ClinicalTrials.gov registry under #NCT00120770 http://clinicaltrials.gov/ct2/show/NCT00120770

## Background

Lower-than-expected incidence of HIV in a HIV prevention trial may undermine sample size calculations and compromise study power [1]. Even if the study community has been well characterized before the trial, the study population will be a selected sub-sample of that community, which may have lower incidence. Furthermore, ethically warranted risk reduction procedures may lead to behavioral changes and increased condom use and thus, lower risk of HIV among the study cohort. This phenomenon, although based on self-reports, has been noted in several trials [2,3].

We conducted a trial to evaluate the efficacy of cellulose sulfate (CS) for preventing HIV in Nigeria. In designing our trial, we based our estimate of HIV incidence on the national HIV prevalence in Nigeria reported two years before study initiation [4-6], and on the incidence-to-prevalence ratio of 2.4 from previous research conducted by Family Health International (FHI) among similar study populations in West Africa [7]. Because we were concerned about the applicability of these data sources to our specific study population, we planned to monitor the HIV infection rate quarterly and to adjust recruitment strategies and sample size in order to protect requisite study power. Below we describe our methodology and results.

## Methods

We conducted a Phase III, randomized, placebo-controlled trial in Lagos and Port Harcourt, Nigeria between November 2004 and March 2007. Three Institutional Review Boards approved the study: those of the College of Medicine, University of Lagos; the University of Port Harcourt Teaching Hospital; and Family Health International. All participants signed written informed consent forms before enrollment. The study was monitored by a Data Monitoring Committee.

Historical data were used to justify our sample size calculations. Roddy et al. [7] reported a prevalence of HIV of 17% and a subsequent incidence of HIV of 7 per 100 woman-years (WY) among sex workers in Cameroon in the late 1990's. In urban areas of Nigeria in 2002 the UNAIDS reported a prevalence of HIV of 5.8% among pregnant women between the ages of 20 and 24, and of 30.5% among female sex workers [4]. From this we conservatively estimated that the prevalence of HIV among high-risk populations in urban Nigeria should not be lower than 12% (i.e., at least twice as high as the prevalence among the antenatal population). Based on the prevalence to incidence ratio of 2.4 observed in the Roddy study, we anticipated an incidence of 5 per 100 WY among placebo users in our trial. Enrolling 2160 women, and following each for one year, was expected to achieve the 66 HIV infections necessary to provide 80% power detect a halving of the incidence rate among CS users (e.g. from 5 to 2.5 per 100 WY). Hence, we monitored the pooled incidence of HIV during the study with the aim of achieving an overall rate of 3.75 per 100 WY.

The admission criteria and study procedures for this study have been described elsewhere [8]. Briefly, a total of 3619 women were screened and 1644 enrolled into the CS trial at the Ikeja and Apapa clinics in Lagos, and at the Central and Peripheral clinics in Port Harcourt. The Ikeja and Apapa clinics screened 598 and 1371, respectively, and enrolled 279 and 584 women into the study. The Central and Peripheral clinics screened 1044 and 605, respectively, and enrolled 518 and 262 women into the study. All enrolled women were at high risk of HIV acquisition, meaning that they reported three or more acts of intercourse per week and more than one sexual partner in the last three months. Most study participants were low-income women who exchanged sex for money to supplement their incomes, although most did not identify themselves as sex workers. We did not exclude professional sex workers from the trial but intentionally limited their number due to the concerns associated with their high coital frequency and its potential impact on gel compliance. Participants were randomized to use CS gel and condoms or placebo gel and condoms for all acts of sexual intercourse throughout the 12 months of study participation. During monthly follow-up visits the participants were tested for HIV, gonorrhea and chlamydial infection.

Study data were collected at the sites and entered by local staff into FHI's database. Blinded datasets were used to assess recruitment strategies one year after study initiation and quarterly, thereafter. We assessed the following parameters, aggregated over the treatment groups: number of screened and enrolled participants, prevalence and incidence of HIV and other sexually transmitted infections (STIs) (by month and overall), demographic characteristics and self-reported sexual behavior. HIV/STI prevalence was defined as a proportion of infected participants among women screened for the study. HIV/STI incidence was defined as the rate of new infections in the study. The site investigators were advised on their recruitment strategies based on the findings of the interim analyses.

## Results

**Figure 1:**
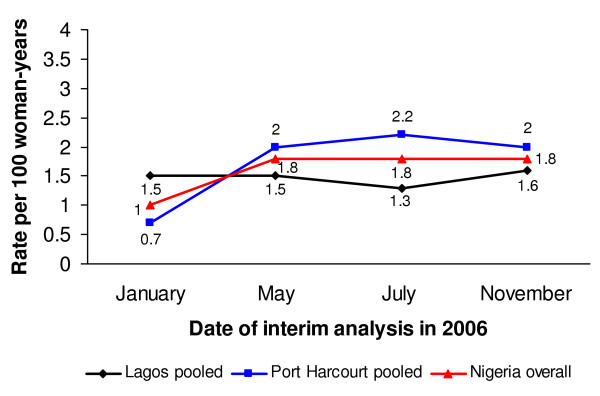
**Incidence of HIV in Nigeria, by site and overall**.

**Figure 2:**
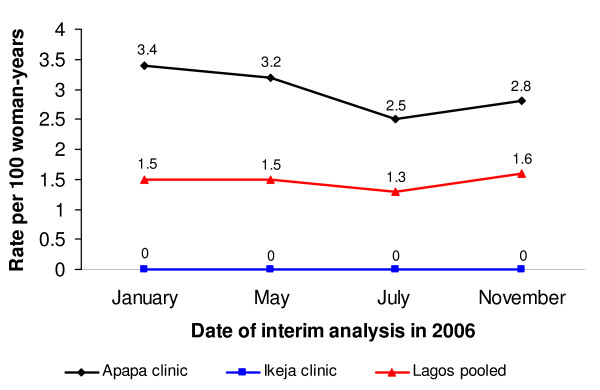
**Incidence of HIV in Lagos, by clinic and overall**.

**Figure 3:**
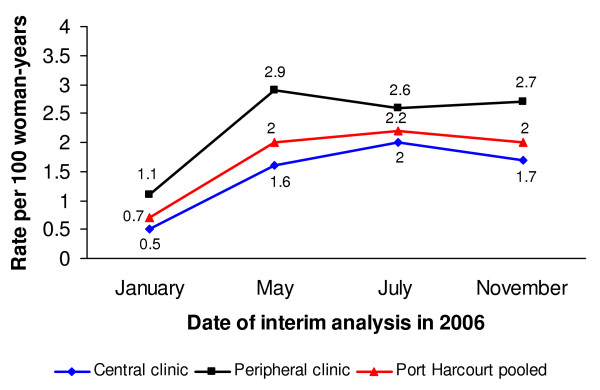
**Incidence of HIV in Port Harcourt, by clinic and overall**.

A total of four such interim analyses were conducted between the initiation of the study in November 2004 and its closure in January 2007: on January 17, 2006 (i.e. approximately one year after study initiation); May 02, 2006; July 31, 2006; and November 21, 2006. The first review demonstrated that the overall incidence of HIV was less than one-third of the pre-study estimate (Figure 1), with only one clinic (the Apapa clinic in Lagos) reporting near the expected rate of 3.4 per 100 WY (Figure 2 and 3).

We noted that although HIV prevalence at screening was highest at the Apapa clinic, women screened at that site had substantially lower coital frequency, fewer partners, and fewer acts unprotected by condoms than women screened at either of the two Port Harcourt clinics, where HIV prevalence was lower (Table 1). We observed a similar inverse association between the prevalence of gonorrhea at screening and self-reported measures of high risk sexual activity. Further, both the HIV prevalence and the sexual activity level in the screened population were lowest at the Ikeja clinic.

**Table 1:** HIV/STI rates and sexual behavior characteristics by clinic, all women screened (January 2006)

Prevalence/characteristic	Apapa	Ikeja	Central	Peripheral
HIV prevalence (%, 95% CI)*	20.1 (17.2, 23.2)	8.7 (6.5, 11.4)	14.3 (11.9, 16.9)	17.3 (13.8, 21.3)

Gonorrhea prevalence (%, 95% CI)*	14.9 (12.2, 18.0)	6.7 (4.6, 9.4)	3.4 (2.2, 5.0)	5.5 (3.5, 8.2)

Chlamydia prevalence (%, 95% CI)*	5.4 (3.7, 7.5)	5.3 (3.4, 7.7)	2.9 (1.8, 4.3)	2.2 (1.0, 4.2)

Average number of coital acts in last week	3.9	2.8	7.1	5.6

Average number of new partners in last month	1.8	0.4	8.2	5.1

Proportion of coital acts protected by condom in last week (%)	66.7	52.0	55.2	59.3

*cumulative (i.e., proportion of infections among all participants screened into the study by January 2006)

The most noticeable differences in demographic characteristics were that women in the Apapa clinic were slightly younger and less educated than those in Ikeja and the two Port Harcourt clinics (Table 2).

**Table 2:** Selected demographic characteristics by clinic, all women screened (January 2006)

Characteristics (%)	Apapa	Ikeja	Central	Peripheral
Age < 25 years	72.9	66.7	71.8	70.6

Years of school <= 12	96.7	87.4	62.7	57.7

Current occupation: student	9.3	23.0	36.4	29.0

Based on the observations at the first interim analysis, we stopped recruiting at Ikeja because of the comparatively low prevalence of HIV and lack of any incident cases during the first year of the study. Similarly, efforts of the recruitment team in Port Harcourt were concentrated on identifying new catchment areas with populations at higher risk of HIV.

Subsequent quarterly analyses showed that HIV prevalence remained relatively high in the Apapa clinic and the two clinics in Port Harcourt, ranging between 14% and 20%. The observed incidence in Lagos decreased slightly between January 2006 and November 2006, even after dropping the Ikeja clinic (Figure 2). The observed HIV incidence in Port Harcourt more than doubled during the same period, however (Figure 3). Seemingly as a result of the changes in recruitment efforts, the overall incidence of HIV in the study increased from 1.0 per 100 WY in January 2006 to 1.8 per 100 WY in November 2006 (Figure 1). Nonetheless, the incidence was noticeably lower than expected when designing the trial, leading the study team to stop enrollment in Nigeria and to expand the trial to South Africa, a country with a much higher background HIV incidence. However, the sites in South Africa never started recruitment because of the premature termination of the study on 31 January 2007 due to safety concerns raised in a parallel CS trial [9].

## Discussion

Accurate incidence estimates are required to make informed sample size and power calculations. The best method of collecting incidence data is to conduct prospective research on large cohorts [10,11]. Such studies are capable of simulating a trial setting, but are time-consuming and extremely expensive. In addition, there is no guarantee that incidence will be maintained once the actual trial starts. As an alternative, laboratory assays can distinguish recent from long-term HIV-1 infections in cross-sectional survey [12]. While this method is quick and inexpensive, it does not simulate a trial setting in terms of recruitment, follow-up, or intervention, and its accuracy has been questioned [13].

Many first-generation HIV prevention trials relied on available country-specific HIV prevalence data and HIV prevalence-incidence ratio from previous trials [2,3,14]. In our study in Nigeria, we estimated that the incidence of HIV should not be lower than 5 per 100 WY in the control arm, and 3.75 per 100 WY overall if the product was 50% effective. Although we considered these assumptions conservative, the trial proved otherwise. The on-going monitoring of the HIV data showed that while most of the clinics reported the expected prevalence of 12% or higher, only one out of four reported an infection rate higher than 3.0 per 100 WY, with an overall incidence of 1.8 per 100 WY.

Our *a priori *data monitoring plan led us to calculate interim incidence rates on a quarterly basis and modify our recruitment strategies accordingly in order to increase the likelihood of enrolling high-risk populations and achieve the target number of events within a reasonable timeframe. The closure of the Ikeja clinic in Lagos and inclusion of new catchment areas in Port Harcourt was associated with an almost two-fold increase of the HIV incidence during the second year of the trial. As a direct result of our efforts we identified pockets of acute HIV transmission and populations at very high risk of HIV in two large cities in Nigeria. For comparison, Padian et al. reported an HIV incidence of 2.7 per 100 WY in Zimbabwe [15], a country where the HIV epidemic is more generalized and national adult HIV prevalence exceeds 15% [16].

Unfortunately, slow enrollment and poor study retention were downsides of our recruitment efforts in Nigeria. It took almost two years to accomplish 75% of the targeted enrollment, instead of the one year originally planned. About one third of the study participants were lost. The participants in the Apapa clinic who were at highest risk of HIV, consistently reported the highest rates of loss to follow-up and missed visits during the study (data not shown). The observed correlation between risk of HIV and study retention suggested that demographics played a major role in loss to follow-up. We compromised our retention rates by recruiting young women at highest risk of HIV infection, residential mobility, and poor compliance with the protocol procedures. While the problem of lower-than-expected incidence can be solved, among other means, by increasing sample size without threatening validity of the results, the consequences of poor retention on interpretation of the data are difficult to overcome. Difficult and prolonged enrollment as well as potential detriment to study retention are serious limitations of conducting a prevention trial among high-risk populations in a low-prevalence country and should be taken into careful consideration before choosing a country and/or site for future HIV-prevention trials.

The observed inverse association between the prevalence of HIV and self-reported measures of sexual activity among the two clinics in Port Harcourt and the Apapa clinic in Lagos could have several explanations. Additional information collected informally by the site investigators in Port Harcourt suggested that recruitment in brothel-like structures and hotels, in many of which the use of condoms was mandatory, was one possible reason for the low HIV rates despite high sexual activity at this site. Seemingly, women in these hotels practiced safe sex and, as a result, were not at high risk of HIV in spite of their high level of sexual activity. However, the self-reported data on condom use does not support this speculation (Table 1). Another possible explanation is that the level of the HIV epidemic in Port Harcourt in general was lower than in the Apapa area in Lagos.

While the interim data monitoring and on-going modification of recruitment strategies appeared to be successful in increasing the overall incidence of HIV, the early closure of the CS trial in Nigeria limited our ability to thoroughly evaluate the effectiveness of this approach. Clearly, the closure of the Ikeja clinic in Lagos was the single most important contributing factor in the increase of the observed overall HIV incidence. The effect would have been more profound if this decision had been made sooner. At the same time it is unclear to what extent the modification of recruitment strategies in Port Harcourt contributed to the increase of the observed overall HIV incidence, or if it would have resulted in further substantial increase if the trial had continued as planned.

## Conclusion

In planning future HIV prevention trials, researchers should use all available means of accurate incidence estimation and, if possible, measurement of HIV incidence in a potential study population. However, given the limitations of the existing methods, a close monitoring of HIV prevalence and incidence rates during the trial seems prudent. The on-going modification of recruitment strategies based on the regular analysis of HIV rates, although not without limitations, appeared to be an efficient method for targeting populations at greatest risk of HIV infection in Nigeria. Direct access to study data and early initiation of regular interim monitoring are important for producing measurable results within a limited time frame.

## Competing interests

The authors declare that they have no competing interests.

## Authors' contributions

VH designed the study, provided overall supervision of its implementation and wrote the paper. FO OKO implemented the study. NM JUO SO acquired and facilitated management of the data. VH FO OKO CW analyzed and interpreted the data. FO OKO NM CW critically revised the paper for important intellectual content. All authors reviewed, approved and take responsibility for the manuscript.

## Pre-publication history

The pre-publication history for this paper can be accessed here:

http://www.biomedcentral.com/1471-2288/9/44/prepub
